# Multilevel determinants of child development in socially vulnerable Brazilian children: a longitudinal study

**DOI:** 10.3389/fped.2025.1675611

**Published:** 2026-01-12

**Authors:** Aline Pereira da Silva, George Oliveira Silva, Marta Rovery de Souza

**Affiliations:** 1 Federal Institute of Goiás, Águas Lindas, Brazil; 2 Faculty of Nursing, Federal University of Goiás, Goiânia, Goiás, Brazil; 3 Institute of Tropical Pathology and Public Health, Federal University of Goiás, Goiânia, Brazil

**Keywords:** child, child development, vulnerable populations, primary health care, longitudinal analysis

## Abstract

Child development consists of a process that involves multiple factors interrelated to the acquisition of children's skills in different areas, such as language, motor, cognitive, emotional, and social. Although extensively explored in literature, there is a gap in how vulnerabilities faced by families can influence this outcome. Thus, this study aims to identify social, maternal, obstetric and familiar determinants of child development in vulnerable children. This is a longitudinal study, conducted in three evaluations between August 2018 and January 2022, with families from Central Brazil participating in the research Impact Evaluation of the Happy Child Program. Generalized Estimating Equations models were used to identify the determinants of child development, evaluated in this study by the Ages and Stages Questionnaire 3rd edition, comparing with sociodemographic, social support, obstetric, children's birth and growth, and family situation variables. Of the 320 families that participated in the study in the state of Goiás, 284 met the inclusion criteria. In general, at the 3 assessment times, the children presented good child development scores in all domains. Positive determinants of child development included growth monitoring and non-violent discipline, while negative determinants were maternal age between 30 and 39 years old and psychological aggression. The findings of the present study highlight critical areas for targeted interventions in child development, offering valuable insights into research and policy formulation for children living in vulnerability situations.

## 1 Introduction

Child development consists of a process that involves multiple factors, interrelated to the acquisition of children's skills in different areas, such as language, motor, cognitive, emotional, and social (1). Although genetics plays an important role in this process, development is also associated with the stimuli and environment in which the child lives (2, 3). In low and middle-income countries, it is estimated that approximately 250 million children under 5 years fail to reach their expected development potential (4), specially related to systemic factors such as low birth weight, high maternal and under-5 mortality (5). In Brazil, the prevalence of developmental delay in preschool children living in an urban area was 25.4% (6).

The World Health Organization (WHO) stablish that it is a human right and a fundamental requirement for sustainable development to ensure ample development opportunities for children (7). Furthermore, ensuring access to quality early childhood development is included in the fourth sustainable development goal for up to 2030 (3).

The ideal environment that contributes to adequate child development is characterized by effective interactions with adults, through stimulation, protection, and attention (2, 3). An environment that provides children with the necessary stimuli generates positive impacts throughout life (3). On the contrary, when exposed to acts of abuse, mistreatment, and neglect, children may experience short- or long-term consequences, such as problems related to health, development, psychosocial problems, and learning (8–10).

Developmental delay is characterized when a child does not perform certain skills at the same level as others of the same age, considering the characteristics of each population (7). Thus, a child's development can be influenced by socioeconomic and family factors, type of parenting, nutrition, and mental health (5). Prematurity and low birth weight are factors that contribute to changes in child development (11). Some authors also point out other factors that can influence child development, such as lower parental education, low family income, poor sanitation conditions, living with a high number of adults and children that does not belong to familiar nuclei in the same household, and the level of interaction between the family and the child (5, 12).

Understanding the determinants of child development in vulnerable populations is crucial for addressing disparities in early childhood outcomes, especially considering the impact of Covid-19 pandemic (13). Vulnerable children, often exposed to socioeconomic, environmental, and health-related adversities, are at heightened risk of developmental delays, which can have long-term consequences on their educational attainment, social integration, and overall well-being (14). Identifying the key factors that influence their development allows for the formulation of targeted interventions that can mitigate these risks and promote equitable growth opportunities. Despite the growing recognition of the importance of early childhood development, there is still limited knowledge regarding the specific determinants that most significantly impact vulnerable children.

This study can help to fill this gap by providing evidence-based insights that can inform policies and programs aimed at fostering healthy development in this high-risk group. Thus, the aim of this study is to identify social, maternal, obstetric and familiar determinants of child development in vulnerable children.

## 2 Methods

### 2.1 Study design

This is a longitudinal study, conducted in three moments between August 2018 and January 2022, with families from Central Brazil participating in the research *Impact Evaluation of the Happy Child Program* (in Portuguese: *Programa Criança Feliz*—PCF). The reporting of this study followed the recommendations of the Strengthening the Reporting of Observational Studies in Epidemiology (STROBE) Statement: guidelines for reporting observational studies (15). The PCF is a program of the Ministry of Citizenship that monitors the child development of children aged 0–6. The program aims to promote the integral development of children, considering the context of the family's life (16).

The main study consisted in a multicenter randomized controlled trial conducted to evaluate the impact of family stimuli in cognitive and psychomotor children development in early childhood, comparing a group of children/families that received training for stimulation (experimental) with a group that did not receive the training (control). The intervention consisted in home visits conducted by advanced professionals who guide parents on childcare, health, and development. The program integrates social assistance, health, education, and cultural policies to strengthen family bonds and child development. The detailed methodology description of the main project is presented in Santos et al. (16) The present study is focused on families of both experimental and control groups from three cities in the state of Goiás (Águas Lindas de Goiás, Luziânia and Novo Gama) that participated in the multicenter study.

### 2.2 Sample

The “longpower” statistical package of R (Iddi & Donohue, 2022) was used to estimate the necessary sample, considering three time points. Thus, 178 participants were estimated to participate in the study, considering a statistical power of 80% (*β* = 20%), a significant level of 95% (*α* = 0.05), and a precision of 3%. As inclusion criteria in the main study was established mothers or caregivers/responsible for children under 12 months of age, beneficiaries of the Bolsa Família Program and eligible for the PCF. In the baseline evaluation 320 children/families living in three cities in the state of Goiás and children between 5 and 11 months of age were included in the study. Considering that this study also evaluates the impact of maternal depression in children's development, only mothers were included, being the final sample consisting of 284 children/families.

### 2.3 Data collection

In the state of Goiás, six interviewers conducted home visits in the three data collection cycles using tablets to record the information. Data was collected by trained health professionals who underwent standardized training sessions before fieldwork to ensure uniform administration of the instruments and to minimize interviewer bias. Training included orientation on the objectives of the study, ethical conduct, and standardized procedures for administering and explaining each developmental milestone assessed in the questionnaire. When needed, interviewers provided simple and culturally appropriate examples to help caregivers understand each item. All field staff were supervised throughout the data collection period to ensure adherence to protocol. The questionnaires were administered to the children's mothers by interviewers trained in a standardized application of the instrument. Up to three contact attempts were standardized to schedule a visit with each family. The interviews lasted an average of 40 min.

**Figure 1:**
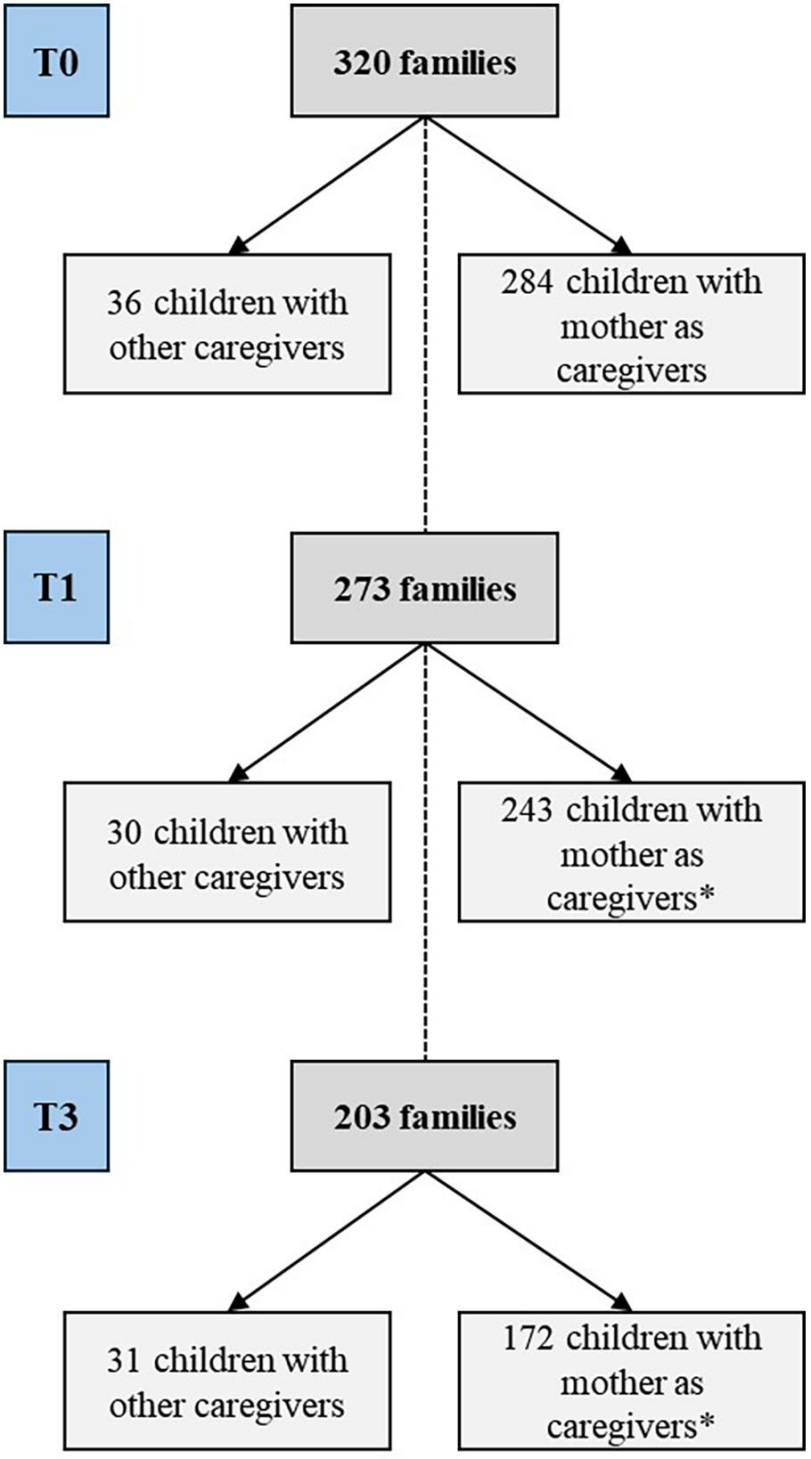
Flowchart of the organization of participants for the study at each moment. Goiás, Brazil, 2018–2022. *Reduction in the number of families due to refusal or because they were not found at the addresses and/or by telephone contact.

The baseline survey (T0) was conducted from August 2018 to July 2019, encompassing 284 children and their families residing in the three cities included in this study that met the inclusion criteria, specifically targeting children aged under 12 months. The first follow-up survey (T1) occurred between September 2019 and January 2020, involving 243 children and families, with the children's ages ranging from 17 to 30 months. As this was the pandemic period, T2 was carried out entirely via telephone, so no data related to the outcome of this study was collected. Although data from T2 was not considered to be included in this analysis, it is important to mention that from T1 to T3 there was an important follow-up loss. The third follow-up survey (T3) took place from November 2021 to January 2022, including 181 children and families, during which the children were aged between 41 and 54 months (Figure 1).

### 2.4 Dependent variable

The dependent variable of this study is the child development, measured by the Ages and Stages Questionnaire, 3rd edition (ASQ-3), instrument used as a parent reported general development screening tool. The AQS-3 is a 30-item instrument with target questions for each age group, developed and validated by Squires and Bricker (17, 18), and translated and validated to Brazilian Portuguese by Filgueiras et al. (19), that can be completed by parents between 12 and 18 min. The instrument evaluates the developmental milestones for children from 2 to 60 months in five domains: (I) Communication, (II) gross motor, (III) fine motor, (IV) problem solving, and (V) personal-social. The ASQ-3 comprises a series of 21 questionnaires for infants (2, 4, 6, and 8 months of age), toddlers (9, 10, 12, 14, 16, 18, 20, 22, 24, 27, 30, and 33 months of age), and preschoolers (36, 42, 48, 54, and 60 months of age) with six question each domain, which indicates the child's achievement of each skill considering the following score: 0 points = not yet; 5 points = sometimes; and 10 points = yes. Each domain has a score ranging from 0 to 60 points. The sum of the responses makes up the score for each domain, considering the child's age group. The sum of all domains was analyzed as the total ASQ-3 score. The instrument was validated in Brazil with children of different social strata, obtaining good internal consistency in all domains (*α* > 0.65), with only 8% of the items demonstrating adjustment problems (19).

### 2.5 Independent variables

Independent variables were grouped in sociodemographic variables, social support, obstetric variables, variables related to children's birth and growth, and family situation at the time of data collection. Sociodemographic variables were defined as maternal and paternal age in years (≤19, 20–29, 30–39, ≥40); maternal and paternal education in years (0–4, 5–8, ≥9); child's age range (T0: <1 year old, 1 year to 1 year and 11 months; T1: 1 year to 1 year and 11 months, 2 years to 2 years and 11 months; T3: 3 years to 3 years and 11 months; ≥4 years old).

Social support was composed by the variables father's support (yes/no), family support (yes/no), lives with partner (yes/no), lives with other family members (yes/no), and live with other people (yes/no). Obstetric variables were composed by living children (up to 1, between 2 and 3, ≥4), planned pregnancy (yes/no), number of prenatal consultations (0–5, ≥6), first prenatal consultation trimester (first, second, third), and type of delivery (vaginal/surgical). Variables related to children's birth and growth were prematurity (yes/no), birth weight in grams (≤2,500, >2,500 and ≤4,000, >4,000), breastfeeding (yes/no), and growth monitoring (yes/no).

Variables related to family situation at the time of data collection were mother/father works outside the home (yes/no), and family income (up to 1 minimum wage/above 1 minimum wage). Maternal depression (yes/no) was measured by the Edinburgh Maternal Depression Scale (EPDS), a 10-item instrument validated for women in the postpartum period (20) and general population (21), that evaluates depressive symptoms in the seven days prior to the interview. A cutoff ≥10 points was used to define the presence of maternal depression. Family conflicts in the parent-child relationship (yes/no), was measured by the Conflict Tactics Scales: Parent-child Version (CTSPC) (22), 22-item instrument used to identify family conflict in four dimensions: nonviolent discipline (NVD), psychological aggression (PSY), corporal punishment (CP), and physical maltreatment (PM).

### 2.6 Statistical analysis

Initially, descriptive analyses of sociodemographic, obstetric, child-related and family situation variables were performed at the time of data collection at the three assessment times and presented in the form of absolute and relative frequencies. The child's development was analyzed according to the ASQ domains (communication, gross motor coordination, fine motor coordination, problem-solving, personal-social) in addition to the total score, defined as the outcome variable of the study. The means and standard deviations of the child's development assessed by the ASQ and its domains at the three assessment times of the study (T0, T1 and T3) were estimated.

In the analysis of the factors associated with development, linear regressions of Gaussian distribution were used from Generalized Estimating Equations (GEE) models (23, 24). The time variable (T0, T1 and T3) was used to define the clusters used in the models. Thus, the models presented 3 clusters with 284 observations and were constructed assuming an exchangeable correlation structure, considering that the correlations between repeated measures within each cluster were assumed to be constant. This structure was statistically supported by presenting the lowest Quasi-Likelihood under the Independence Model Criterion (QIC) compared to other tested structures (25). Maternal, paternal and child sociodemographic characteristics, obstetric characteristics, characteristics related to social support and child growth, in addition to the family situation at the time of collection were established as independent variables.

Initially, bivariate analyses were performed to investigate the association between the study outcome measure and each independent variable analyzed. Then, variables with *p*-values < 0.20 were included in a hierarchical multiple linear regression model with robust variance estimation. Multiple regression analysis was performed using three models based in the conceptual model (26) defined in a previous study of the main project (27) and adapted for this study, so that potential fixed factors associated with development were classified into hierarchical levels: distal factors (sociodemographic characteristics), intermediate factors (characteristics related to social support and childbirth, birth, and growth), and proximal factors (characteristics of current family life, maternal depression, and family conflicts).

The presence of multicollinearity was assessed by inspecting the variance inflation factors (VIF) and the condition number (*κ*) derived from the predictor matrix of the GEE model, considering multicollinearity to be high when VIF > 5 or *κ* > 30 (28). The variables from the initial models, even when they did not present statistical significance, were maintained for adjustment in subsequent models, as they are potentially confounding variables. Model 1 consisted of family sociodemographic variables (maternal and paternal age and education, and child's age at the time of data collection). Model 2 included the variables from model 1 and variables related to social support, obstetric variables, and variables related to childbirth, birth, and child growth (family support, trimester of the first prenatal consultation, birth weight, and prematurity). In model 3, in addition to the previous variables, the family characteristics at the time of the interview (maternal depression, non-violent discipline and psychological aggression) were included.

In all models, the variable participation in the PCF (yes/no) was included as an adjustment variable. Missing values were treated using the Multiple Imputations by Chained Equations (MICE) imputation method, estimated using the R “mice” statistical package (29), considering that the variables presented between 10% and 30% of missing values. The values of the model coefficients (*β*) and the robust standard error (SE) values, which represent the correlation structure between the observations, were extracted. Values of *p* < 0.05 were considered statistically significant. To quantify the magnitude of associations in the multiple GEE models, we computed standardized effect sizes using the “RESI” R package, based on model coefficients and the marginal dispersion parameter. The statistical analysis was performed using the R statistical program (R Core Team, Vienna, Austria) version 4.4.1, and the “geepack” was used to perform the GEE analyses (30).

### 2.7 Ethical aspects

The Research Ethics Committee of the Federal University of Pelotas approved the *Impact Evaluation of the Happy Child Program* project, with approval number 2,148,689.

## 3 Results

### 3.1 Participants' characteristics

Of the 320 families that participated in the study in the state of Goiás, 284 met the inclusion criteria. Table 1 presents the characteristics of the children/families participating in the study. Regarding maternal and paternal sociodemographic characteristics, there was a predominance of mothers and fathers between 20 and 29 years of age, and with more than 9 years of education. Most mothers reported having support from the father and family, while the mothers who lived with their partners and children were the predominant family dynamics. A small portion reported living with other family members or other people.

**Table 1:** Characteristics of families/children participating in the impact evaluation of the happy child program project during the three evaluation periods. Goiás, Brazil, 2018–2022.

Variables	T0 (*n* = 284)		T1 (*n* = 243)		T3 (*n* = 181)	
	*n*	%	*n*	%	*n*	%
Sociodemographic variables						
*Maternal*						
Age						
≤19 years old	47	16.6	21	8.6	4	3.2
20 to 29 years old	132	46.6	118	48.6	85	68.0
30 to 39 years old	92	32.5	91	37,4	12	9.6
≥40 years old	12	4.2	13	5.3	24	19.2
Education (in years)						
0 to 4 years	27	10.4	26	11.7	14	8.3
5 to 8 years	62	23.8	50	22.4	34	20.1
≥9 years	171	65.8	147	65.9	121	71.6
*Paternal*						
Age						
≤19 years old	7	2.5	5	2.1	3	1.7
20 to 29 years old	134	48.4	112	47.3	75	42.6
30 to 39 years old	92	33.2	83	35.0	67	38.1
≥40 years old	44	15.9	37	15.6	31	17.6
Education (in years)						
0 to 4 years	36	14.8	34	16.5	20	13.0
5 to 8 years	75	30.9	60	29.1	49	31.8
≥9 years	132	54.3	112	54.4	85	55.2
*Child*						
Child's age						
Age range 1	202	71.4	170	70.0	124	68.5
Age range 2	81	28.6	73	30.0	57	31.5
Social support						
Father's support						
Yes	250	89.3	214	88.8	156	86.7
No	30	10.7	27	11.2	24	13.3
Family support						
Yes	260	92.9	222	92.1	166	92.7
No	20	7.1	19	7.9	13	7.3
Lives with partner						
Yes	182	75.5	184	75.7	129	76.3
No	59	24.5	59	24.3	40	23.7
Lives with children						
Yes	177	73.4	179	73.7	130	76.9
No	64	26.6	64	26.3	39	23.1
Lives with other family members						
Yes	89	31.7	75	31.0	60	33.3
No	192	68.3	167	69.0	120	66.7
Live with other people						
Yes	10	3.6	9	3.7	6	3.3
No	270	96.4	232	96.3	174	96.7
Obstetric variables						
Living children						
Up to 1	89	42.2	76	42.0	60	43.5
Between 2 and 3	87	41.2	75	41.4	58	42.0
≥4	35	16.6	30	16.6	20	14.5
Planned pregnancy						
Yes	103	36.4	85	35.0	69	38.1
No	180	63.6	158	65.0	112	61.9
Number of prenatal consultations						
≥6	204	73.1	178	74.2	133	74.7
0 to 5	75	26.9	62	25.8	45	25.3
First prenatal consultation trimester						
First	201	75.0	171	74.0	129	75.4
Second	58	21.6	51	22.1	36	21.1
Third	9	3.4	9	3.9	6	3.5
Type of delivery						
Vaginal	190	67.1	167	68.7	127	70.2
Surgical	93	32.9	76	31.3	54	29.8
Variables related to the birth and growth of the child						
Prematurity						
Yes	20	7.1	17	7.1	9	5.0
No	261	92.9	224	92.9	170	95.0
Birth weight (in grams)						
≤2,500	17	6.3	12	5.2	9	5.2
>2,500 and ≤4,000	242	89.6	210	90.9	158	91.9
>4,000	11	4.1	9	3.9	5	2.9
Breastfeeding						
Yes	226	79.9	196	80.7	146	80.7
No	57	20.1	47	19.3	35	19.3
Growth monitoring						
Yes	245	86.6	67	27.6	177	97.8
No	38	13.4	176	72.4	4	2.2
Family situation at the time of data collection						
Mother works outside the home						
Yes	19	6.7	16	6.6	10	5.5
No	264	93.3	227	93.4	171	94.5
Father works outside the home						
Yes	225	82.7	192	82.1	139	80.3
No	47	17.3	42	17.9	34	19.7
Family income						
Up to 1 minimum wage	162	57.2	122	50.2	95	52.5
Above 1 minimum wage	121	42.8	121	49.8	86	47.5
Maternal depression						
Yes	84	29.7	63	25.9	55	32.0
No	199	70.3	180	74.1	117	68.0
*Conflict Tactics Scales: Parent-child Version (CTSPC)*						
Nonviolent discipline (NVD)						
Yes	194	68.6	170	70.0	169	93.4
No	89	31.4	73	30.0	12	6.6
Psychological aggression (PSY)						
Yes	53	18.8	88	36.2	101	55.8
No	229	81.2	155	63.8	80	44.2
Corporal punishment (CP)						
Yes	54	19.1	63	25.9	92	50.8
No	228	80.9	180	74.1	89	49.2
Physical maltreatment (PM)						
Yes	5	1.8	33	13.6	115	63.5
No	277	98.2	210	86.4	66	36.5

Regarding obstetric characteristics, most mothers already had other children and did not plan the pregnancy of the child participating in the study. The majority reported having had more than 6 prenatal consultations and the first consultation occurred in the first trimester of pregnancy. Vaginal delivery was the predominant method. There was a predominance of non-premature children, who were born with adequate weight and who were breastfeeding at the three data collection times. Most children had their growth monitored at T0, verified by the record in the child's health booklet. However, at T1 there was an inversion, while at T3 only 4 children had no monitoring in the last year.

There was a greater predominance of women without depression, but still with a high prevalence. Regarding the family conflicts verified by the CTSPC, there was a predominance of families that used non-violent methods in the three moments. However, in relation to psychological aggression, corporal punishment and physical abuse in T0 and T1 there was a predominance of families that did not use these violent methods, while in T3 there was a predominance of those that used them.

### 3.2 Child development and bivariate analyses

As shown in Table 2, most ASQ-3 domains demonstrated relative stability over the three evaluation periods. In the communication domain, the mean score slightly decreased from 50.0 (SD = 11.0) at T0 to 47.4 (SD = 13.5) at T1, before returning to 50.2 (SD = 11.2) at T3, indicating recovery to baseline levels (*β* = −0.22; *p* = 0.170). The gross motor domain followed a similar pattern, with a modest fluctuation from 45.9 (SD = 12.7) at T0 to 51.4 (SD = 11.7) at T1 and 44.9 (SD = 14.0) at T3 (*β* = 0.19; *p* = 0.230). For fine motor skills, a significant improvement was observed over time, increasing from 51.2 (SD = 11.2) at T0 to 52.2 (SD = 10.6) at T3 (*β* = −0.36; *p* = 0.041), suggesting a positive developmental trajectory in this domain. Conversely, problem-solving abilities showed a small decline, from 49.4 (SD = 12.8) at T0 to 45.0 (SD = 13.0) at T1 and 50.7 (SD = 12.0) at T3, without statistical significance (*β* = −0.34; *p* = 0.144). Scores in the social-personal domain remained stable throughout the study period [47.7 [SD = 11.8] at T0, 46.7 [SD = 11.5] at T1, 47.7 [SD = 12.9] at T3; *β* = −0.20; *p* = 0.250]. Finally, the total ASQ-3 score showed minimal variation, from 243.0 (SD = 41.4) at baseline to 245.0 (SD = 42.9) at T3 (*β* = −0.74; *p* = 0.140). Taken together, these findings indicate overall stability in developmental performance across domains, with only the fine motor domain showing a significant positive change over time.

**Table 2:** ASQ-3 scores of children participating in the impact evaluation of the happy child program project during the three evaluation periods. Goiás, Brazil, 2018–2022.

ASQ domains	T0 (*n* = 284)		T1 (*n* = 243)		T3 (*n* = 181)		Change over time	
	Mean	SD	Mean	SD	Mean	SD	*β*	*p*-value
Communication	50.0	11.0	47.4	13.5	50.2	11.2	−0.22	0.170
Gross motor	45.9	12.7	51.4	11.7	44.9	14.0	0.19	0.230
Fine motor	51.2	11.2	45.2	13.7	52.2	10.6	−0.36	0.041*
Problem-solving	49.4	12.8	45.0	13.7	50.2	12.8	−0.34	0.144
Social-personal	47.7	11.8	46.7	11.5	47.7	12.9	−0.201	0.250
Total score	243.0	41.4	236.0	45.9	245.0	42.9	−0.74	0.140

* *p* < 0.05.

Supplementary Table S1 presents the findings of ASQ-3 according to independent variables. The bivariate analysis showed that the positive determinants of child development were 9 or more years of education (*p* = 0.021), growth monitoring (*p* = 0.032), and nonviolent discipline (*p* = 0.041), while maternal age between 30 and 39 years (*p* = 0.008) and 40 years or more (*p* = 0.024), prematurity (*p* = 0.050), maternal depression (*p* = 0.045) and psychological aggression (*p* = 0.033) were negative determinants (Table 3).

**Table 3:** Bivariate GEE analyses of the children development determinants of children participating in the impact evaluation of the happy child program project. Goiânia, Goiás, Brazil, 2018–2022.

Variables	Total		Communication		Gross motor		Fine motor		Problem solving		Social-personal	
	*β*	*p*-value	*β*	*p*-value	*β*	*p*-value	*β*	*p*-value	*β*	*p*-value	*β*	*p*-value
Sociodemographic variables												
*Maternal*												
Age												
≤19 years old	Ref		Ref		Ref		Ref		Ref		Ref	
20 to 29 years old	−5.85	0.066	−2.52	**0** **.** **026**	1.44	0.250	−1.72	0.193	−2.47	0.094	−1.12	0.316
30 to 39 years old	−11.15	**0** **.** **008**	−2.84	**0** **.** **036**	0.51	0.730	−2.67	0.097	−3.81	**0** **.** **022**	−2.58	0.055
≥40 years old	−14.44	**0** **.** **024**	−3.51	0.135	0.33	0.880	−0.04	0.987	−6.27	**0** **.** **009**	−4.73	**0** **.** **040**
Education (in years)												
0 to 4 years	Ref		Ref		Ref		Ref		Ref		Ref	
5 to 8 years	15.74	0.118	2.15	0.460	1.84	0.520	6.43	**0** **.** **020**	3.33	0.189	1.44	0.500
≥9 years	21.97	**0** **.** **021**	4.05	0.140	0.92	0.740	7.47	**0** **.** **003**	5.21	**0** **.** **024**	3.40	0.080
*Paternal*												
Age												
≤19 years old	Ref		Ref		Ref		Ref		Ref		Ref	
20 to 29 years old	−9.59	0.390	0.01	1.000	−4.49	0.195	−1.67	0.540	−3.01	0.466	−0.96	0.750
30 to 39 years old	−13.53	0.240	−0.69	0.830	−4.11	0.246	−3.43	0.220	−4.50	0.280	−1.50	0.630
≥40 years old	−20.36	0.110	−1.79	0.600	−7.66	**0** **.** **046**	−3.51	0.280	−5.30	0.221	−3.46	0.300
Education (in years)												
0 to 4 years	Ref		Ref		Ref		Ref		Ref		Ref	
5 to 8 years	11.33	0.190	0.89	0.710	0.36	0.880	3.16	0.208	3.63	0.105	2.22	0.310
≥9 years	12.72	0.110	1.76	0.390	0.21	0.920	3.88	0.093	3.51	0.099	2.87	0.160
*Child*												
Child's age												
Age range 1	Ref		Ref		Ref		Ref		Ref		Ref	
Age range 2	−1.63	0.750	−1.52	0.240	0.67	0.600	0.87	0.520	0.09	0.953	−0.41	0.75
Social support												
Father's support												
Yes	2.25	0.760	1.75	0.370	3.65	0.100	−0.07	0.970	−2.86	0.098	0.33	0.860
No	Ref		Ref		Ref		Ref		Ref		Ref	
Family support												
Yes	22.03	0.053	3.70	0.140	2.97	0.350	6.43	**0** **.** **029**	4.46	0.129	5.64	**0** **.** **016**
No	Ref		Ref		Ref		Ref		Ref		Ref	
Lives with partner												
Yes	−3.21	0.570	0.22	0.880	−1.35	0.320	−0.38	0.790	−1.13	0.493	−1.36	0.380
No	Ref		Ref		Ref		Ref		Ref		Ref	
Lives with children												
Yes	−5.25	0.320	−1.00	0.450	−1.59	0.260	−0.01	0.990	−1.49	0.308	−2.26	0.062
No	Ref		Ref		Ref		Ref		Ref		Ref	
Lives with other family members												
Yes	5.82	0.210	2.08	0.084	−1.05	0.440	0.43	0.730	2.63	**0** **.** **040**	1.64	0.180
No	Ref		Ref		Ref		Ref		Ref		Ref	
Live with other people												
Yes	−5.89	0.450	−7.24	**0** **.** **003**	−0.73	0.810	−1.19	0.700	2.66	0.362	−0.10	0.970
No	Ref		Ref		Ref		Ref		Ref		Ref	
Obstetric variables												
Living children												
Up to 1	Ref		Ref		Ref		Ref		Ref		Ref	
Between 2 and 3	−0.02	1.000	0.20	0.854	−1.03	0.520	−0.70	0.650	0.08	0.960	0.87	0.571
≥4	−10.97	0.260	−1.41	0.460	1.12	0.700	−2.81	0.230	−4.66	**0** **.** **048**	−4.44	**0** **.** **047**
Planned pregnancy												
Yes	−0.24	0.960	−0.19	0.870	−0.18	0.890	−0.07	0.950	−0.44	0.742	−0.06	0.960
No	Ref		Ref		Ref		Ref		Ref		Ref	
Number of prenatal consultations												
≥6	Ref		Ref		Ref		Ref		Ref		Ref	
0 to 5	3.32	0.530	−1.76	0.240	0.27	0.860	1.44	0.310	1.52	0.293	1.75	0.170
First prenatal consultation trimester												
First	Ref		Ref		Ref		Ref		Ref		Ref	
Second	−1.33	0.820	−2.48	0.132	−0.66	0.710	0.00	1.000	0.11	0.941	1.18	0.390
Third	−24.11	0.170	−4.58	0.081	−1.96	0.600	−4.72	0.330	−7.32	0.065	−3.94	0.390
Type of delivery												
Vaginal	−7.39	0.120	−1.10	0.360	−1.04	0.450	−1.96	0.120	−2.21	0.098	−0.79	0.540
Surgical	Ref		Ref		Ref		Ref		Ref		Ref	
Variables related to the birth and growth of the child												
Prematurity												
Yes	−13.47	**0** **.** **050**	−0.09	0.970	−1.27	0.590	−6.21	**0** **.** **004**	−3.43	0.062	−3.00	0.200
No	Ref		Ref		Ref		Ref		Ref		Ref	
Birth weight (in grams)												
≤2,500	Ref		Ref		Ref		Ref		Ref		Ref	
>2,500 and ≤4,000	6.26	0.500	−0.40	0.870	4.50	0.128	1.94	0.409	0.88	0.706	−0.76	0.710
>4,000	16.51	0.160	0.26	0.930	8.63	**0** **.** **008**	6.88	**0** **.** **021**	1.48	0.725	−1.69	0.670
Breastfeeding												
Yes	7.47	0.270	1.48	0.380	−0.58	0.730	3.38	0.073	2.51	0.150	1.60	0.350
No	Ref		Ref		Ref		Ref		Ref		Ref	
Growth monitoring												
Yes	6.12	**0** **.** **032**	2.74	**0** **.** **005**	−6.31	**<0** **.** **001**	4.77	**<0** **.** **001**	3.35	**0** **.** **001**	1.23	0.210
No	Ref		Ref		Ref		Ref		Ref		Ref	
Family situation at the time of data collection												
Mother works outside the home												
Yes	Ref		Ref		Ref		Ref		Ref		Ref	
No	−9.21	0.220	−3.93	0.062	−2.65	0.220	−1.96	0.360	−0.19	0.930	−0.92	0.670
Father works outside the home												
Yes	Ref		Ref		Ref		Ref		Ref		Ref	
No	4.58	0.410	0.51	0.740	−1.31	0.400	1.16	0.450	1.79	0.315	2.26	0.097
Family income												
Up to 1 minimum wage	Ref		Ref		Ref		Ref		Ref		Ref	
Above 1 minimum wage	2.58	0.590	0.95	0.430	0.74	0.580	0.53	0.670	1.09	0.406	−0.46	0.710
Maternal depression												
Yes	−6.14	**0** **.** **045**	−2.21	**0** **.** **028**	−0.66	0.530	−1.12	0.290	−1.59	0.185	−1.40	0.180
No	Ref		Ref		Ref		Ref		Ref		Ref	
*Conflict Tactics Scales: Parent-child Version (CTSPC)*												
Nonviolent discipline (NVD)												
Yes	5.73	**0** **.** **041**	0.87	0.330	−0.35	0.680	1.51	0.110	2.18	**0** **.** **039**	2.77	**0** **.** **004**
No	Ref		Ref		Ref		Ref		Ref		Ref	
Psychological aggression (PSY)												
Yes	−5.31	**0** **.** **033**	−1.55	0.080	1.14	0.120	−1.27	0.130	−1.98	**0** **.** **036**	−0.42	0.610
No	Ref		Ref		Ref		Ref		Ref		Ref	
Corporal punishment (CP)												
Yes	0.92	0.700	1.30	0.099	−0.03	0.970	−0.33	0.710	0.44	0.659	0.56	0.470
No	Ref		Ref		Ref		Ref		Ref		Ref	
Physical maltreatment (PM)												
Yes	1.09	0.560	1.77	**0** **.** **005**	−1.88	**0** **.** **007**	0.56	0.460	−0.39	0.611	0.51	0.450
No	Ref		Ref		Ref		Ref		Ref		Ref	

Bold values represents *p* < 0.05.

In the communication domain, growth monitoring (*p* = 0.005) and the absence of physical abuse (*p* = 0.005) were identified as positive determinants of better developmental outcomes. In contrast, paternal age between 20 and 29 years (*p* = 0.026) and 30–39 years (*p* = 0.036), living with other people (*p* = 0.003), and maternal depression (*p* = 0.028) were associated with lower communication scores.

For gross motor coordination, higher birth weight (>4,000 g) was a positive determinant (*p* = 0.008). Conversely, paternal age ≥40 years (*p* = 0.046), lack of growth monitoring (*p* < 0.001), and experience of physical abuse (*p* = 0.007) were associated with poorer performance in this domain. In the fine motor domain, maternal education between 5 and 8 years (*p* = 0.020) and ≥9 years (*p* = 0.003), family support (*p* = 0.029), birth weight >4,000 g (*p* = 0.021), and growth monitoring (*p* < 0.001) were positive determinants. Prematurity, however, was negatively associated with fine motor development (*p* = 0.004).

For problem-solving, positive determinants included maternal education ≥9 years (*p* = 0.024), living with other family members (*p* = 0.040), growth monitoring (*p* = 0.001), and non-violent discipline (*p* = 0.039). On the other hand, maternal age between 30 and 39 years (*p* = 0.022) or ≥40 years (*p* = 0.009), having four or more children (*p* = 0.048), and exposure to psychological aggression (*p* = 0.036) were negative determinants. In the social-personal domain, family support (*p* = 0.016) and non-violent discipline (*p* = 0.004) positively influenced development, while maternal age ≥40 years (*p* = 0.040) and having four or more children (*p* = 0.047) were associated with lower scores.

### 3.3 Multilevel determinants of child development

The first multiple regression model, including the distal determinants, showed that only mothers' age between 30 and 39 years old remains significant (*β* = −9.03; *p* = 0.037; *d* = −0.12) as a negative determinant of child development. The second model, including distal and medial determinants, showed that growth monitoring (*β* = 5.85; *p* = 0.047; *d* = 0.12) remains as positive determinant. The third model, including distal, medial and proximal determinants, showed that nonviolent discipline (*β* = 5.65; *p* = 0.032; *d* = 0.12) remains as a positive determinant and psychological aggression (*β*=-5.46; *p* = 0.023; *d* = −0.13) remains as a negative determinant (Table 4).

**Table 4:** Multiple GEE regression models of the children development determinants of children participating in the impact evaluation of the happy child program project. Goiânia, Goiás, Brazil, 2018–2022.

Variables	Beta (*β*)	SE robust	*p*-value
***Model 1***			
**Participation in the PCF**			
Yes	3.46	5.49	0.529
No	Ref		
**Sociodemographic variables**			
*Maternal*			
Age			
≤19 years old	Ref		
20 to 29 years old	−5.38	3.18	0.091
30 to 39 years old	−9.04	4.33	**0** **.** **037**
≥40 years old	−10.64	6.80	0.118
Education (in years)			
0 to 4 years	Ref		
5 to 8 years	13.67	10.61	0.197
≥9 years	19.27	10.75	0.073
*Paternal*			
Age			
≤19 years old	Ref		
20 to 29 years old	−3.63	11.20	0.746
30 to 39 years old	−4.50	11.75	0.702
≥40 years old	−4.84	13.02	0.710
Education (in years)			
0 to 4 years	Ref		
5 to 8 years	5.33	7.62	0.484
≥9 years	3.08	7.36	0.676
***Model 2***			
**Social support**			
Family support			
Yes	19.04	9.97	0.056
No	Ref		
**Obstetric variables**			
First prenatal consultation trimester			
First	Ref		
Second	−1.41	5.17	0.784
Third	−21.51	16.21	0.185
Type of delivery			
Vaginal	−8.04	4.85	0.097
Surgical	Ref		
**Variables related to the birth and growth of the child**			
Prematurity			
Yes	−10.67	7.86	0.175
No	Ref		
Birth weight (in grams)			
≤2,500	Ref		
>2,500 and ≤4,000	6.65	9.40	0.479
>4,000	16.43	12.60	0.192
Growth monitoring			
Yes	5.70	2.87	**0** **.** **047**
No	Ref		
***Model 3***			
**Family situation at the time of data collection**			
Maternal depression			
Yes	−4.55	2.96	0.124
No	Ref		
*Conflict Tactics Scales: Parent-child Version (CTSPC)*			
Nonviolent discipline			
Yes	5.78	2.69	**0** **.** **032**
No	Ref		
Psychological aggression			
Yes	−5.52	2.43	**0** **.** **023**
No	Ref		

## 4 Discussion

The findings indicate a complex interaction among distal, medial, and proximal factors impacting child development. In the first model, only mothers' age between 30 and 39 years old emerged as a significant negative determinant, which remained in the second model, in addition to growth monitoring. The negative association between advanced maternal age and child development may reflect the influence of biological, psychosocial, and contextual factors. Socioeconomic profile, maternal mental health, and social support are crucial determinants of the outcome, and can reverse or accentuate risks traditionally attributed to maternal age (31–33), as is the case in this study which included only families in situations of social vulnerability.

The positive effect of growth monitoring may highlight the importance of regular health surveillance in promoting early identification and intervention for developmental delays (34–36). This finding supports the role of healthcare access and consistent monitoring in child development. However, the inverse relationship with maternal age might reflect lifestyle or socio-economic factors associated with older maternal age, such as increased parenting stress or competing responsibilities, that could affect child development indirectly (37). Understanding how these factors intersect can be fundamental in guiding interventions that support mothers across age groups.

The final model highlights the role of proximal factors, such as discipline practices, showing that nonviolent discipline positively and psychological aggression negatively impact development. These findings align with literature that links positive parenting practices with healthier child development outcomes, while negative interactions, like psychological aggression, can impede emotional and cognitive growth (38–40). Findings from worldwide study suggest that 220.4 million (95% CI 138.1, 283.7) and 230.7 million (95% CI 128.4, 300.6) children were exposed to aggressive physical and psychological discipline, respectively (41).

Considering that the global prevalence of nonviolent discipline is 83.9% (41), the results evidence the impact of parenting using these types of discipline methods. Nonviolent discipline strategies may foster a nurturing environment that encourages children to explore and learn without fear, while exposure to psychological aggression could introduce stress that hinders development (40). This model underscores the importance of promoting supportive and nonviolent parenting as critical for fostering optimal child development.

Regarding the factors related to ASQ-3 domains, motor development in children is considered to have phases that can be influenced by risk factors (42). Monitoring growth and development and maternal education over 9 years were associated with increased scores in the domains of fine motor coordination and problem solving. Social support was positively related to good socio-personal development and fine motor coordination. Corroborating these findings, some articles have identified maternal education, family income and social support as factors associated with child development (43–45).

On the other hand, prematurity was the only factor that negatively influenced fine motor coordination. Thus, some studies corroborate the findings, such as two studies conducted in Brazil that identified prematurity and low birth weight as factors that affect child development, considering that restrictions related to these conditions generate more intensive parental care that can contribute to changes in maternal mental health, such as depression, anxiety and consequently unsatisfactory levels of stimulation of the child (46, 47).

Additionally, the lack of public safety, violence, and changes in mental health are considered factors that threaten the child's development (48). The findings show that the non-violent discipline approach with the child was positively related to development in the problem-solving and socio-personal domains. In recent randomized trials implementing the WHO Care for Child Development (CCD) guidelines in Iran, was observed significant improvements only in children's cognitive scores and anxiety/depression subscales at 18 months, suggesting a limited short-term impact of brief group-based interventions (49, 50).

This study has some limitations that should be acknowledged. First, there was considerable attrition between baseline and the third wave (T3), which may have introduced attrition bias and affected the representativeness of the final sample. Although multiple imputation techniques were applied to minimize data loss, it is possible that families who remained in the study differed systematically from those lost to follow-up, potentially leading to an overrepresentation of families with greater stability or engagement in the program. Second, the study relied on maternal self-report instruments, including the ASQ-3, the CTSPC, and EPDS. Although these are validated tools widely used in child development research, self-reported measures are subject to reporting and social desirability bias, particularly when addressing sensitive issues such as parenting practices and maternal mental health.

Finally, the Covid-19 pandemic occurred during the follow-up period (2018–2022), which may have acted as an unmeasured confounding factor. Pandemic-related stressors, social isolation, and disruptions in health and early childhood services could have influenced both maternal well-being and child development trajectories. While data collection at T2 was suspended to mitigate bias, residual confounding cannot be fully excluded. Future studies with post-pandemic cohorts and larger samples are warranted to confirm and expand these findings.

## 5 Conclusion

The study identified a range of characteristics and determinants associated with child development. The majority of participating families had mothers and fathers aged 20–29, with over 9 years of education and predominantly brown-skinned mothers. Most mothers lived with their partners and children, had family and paternal support, and had planned pregnancies. Prenatal care was generally adequate, with most mothers initiating consultations in the first trimester and undergoing vaginal delivery. Children predominantly had healthy birth outcomes and breastfeeding rates remained high across the three assessment periods. Growth monitoring was consistent, although it declined at T1 and improved at T3. Positive determinants of child development included maternal education, growth monitoring, and non-violent discipline, while negative determinants were maternal age ≥30, maternal depression, prematurity, and psychological aggression. Multilevel analysis confirmed non-violent discipline, and growth monitoring as significant positive predictors, and psychological aggression as a consistent negative predictor.

These findings highlight critical areas for targeted interventions in child development, offering valuable insights for research and policy formulation for children living in vulnerability situation. The positive influence of growth monitoring and non-violent discipline underscores the importance of family-centered health strategies and supportive parenting programs. Addressing negative factors like maternal depression and psychological aggression can be pivotal in enhancing developmental outcomes. Policymakers can leverage these insights to design comprehensive maternal and child health policies that prioritize early childhood interventions, particularly for at-risk families. Future research could explore tailored strategies to mitigate adverse determinants and promote equitable access to resources, ensuring that all children achieve their developmental potential.

## Data Availability

The raw data supporting the conclusions of this article will be made available by the authors, without undue reservation.
